# An objective skill assessment framework for microsurgical anastomosis based on ALI scores

**DOI:** 10.1007/s00701-024-05934-1

**Published:** 2024-02-24

**Authors:** Soheil Gholami, Anaëlle Manon, Kunpeng Yao, Aude Billard, Torstein R. Meling

**Affiliations:** 1 Learning Algorithms and Systems Laboratory (LASA), École Polytechnique Fédérale de Lausanne (EPFL), Lausanne, Switzerland; 2 Department of Neurosurgery, The National Hospital of Denmark, Rigshospitalet, Copenhagen, Denmark

**Keywords:** Microsurgery, Skill acquisition, Computer-assisted skill assessment, Objective skill assessment, Quantitative skill assessment

## Abstract

**Introduction:**

The current assessment and standardization of microsurgical skills are subjective, posing challenges in reliable skill evaluation. We aim to address these limitations by developing a quantitative and objective framework for accurately assessing and enhancing microsurgical anastomosis skills among surgical trainees. We hypothesize that this framework can differentiate the proficiency levels of microsurgeons, aligning with subjective assessments based on the ALI score.

**Methods:**

We select relevant performance metrics from the literature on laparoscopic skill assessment and human motor control studies, focusing on time, instrument kinematics, and tactile information. This information is measured and estimated by a set of sensors, including cameras, a motion capture system, and tactile sensors. The recorded data is analyzed offline using our proposed evaluation framework. Our study involves 12 participants of different ages ($35.42\pm 9.78$ years) and genders (nine males and three females), including six novice and six intermediate subjects, who perform surgical anastomosis procedures on a chicken leg model.

**Results:**

We show that the proposed set of objective and quantitative metrics to assess skill proficiency aligns with subjective evaluations, particularly the ALI score method, and can effectively differentiate novices from more proficient microsurgeons. Furthermore, we find statistically significant disparities, where microsurgeons with intermediate level of skill proficiency surpassed novices in both task speed, reduced idle time, and smoother, briefer hand displacements.

**Conclusion:**

The framework enables accurate skill assessment and provides objective feedback for improving microsurgical anastomosis skills among surgical trainees. By overcoming the subjectivity and limitations of current assessment methods, our approach contributes to the advancement of surgical education and the development of aspiring microsurgeons. Furthermore, our framework emerges to precisely distinguish and classify proficiency levels (novice and intermediate) exhibited by microsurgeons.

## Introduction

Microsurgery is primarily used to anastomose small blood vessels (arteries and veins) and to coapt nerves. Microvascular and microneural coaptation permit the complex repair of human tissue after trauma, cancer, and congenital deficiencies, making microsurgical skills essential to several surgical specialties and in various medical procedures, such as cardiac bypass surgery [14], brain bypass surgery [35], intracranial bypass surgery [29], organ transplantation surgery [20], and replantation surgery [17].

Microsurgical operations focus on repairing blood vessels with small diameters, typically less than 3 mm [15, 24], or even smaller (0.3–0.8 mm) in super-microsurgery [19]. Surgeons working at this scale have minimal or no tactile sensation and limited spatial perception, making the procedure challenging. Hence, performing surgery on those vessels presents unique challenges. Any error made during the operation significantly increases the likelihood of vessel occlusion. Hence, it requires extraordinary manual dexterity, in addition to the utilization of stereoscopic vision and visuospatial skills. Despite the importance of skill acquisition in surgical education, the assessment and teaching of this aspect remain the least standardized element in operation rooms.

Four categories were identified by Kalu et al. [13] to classify subjective and objective assessments of microsurgical skills. These are the following: (i) visuospatial ability, (ii) dexterity, (iii) operative flow, and (iv) judgment. In this work, we focus on the first two classes. *Visuospatial ability* is a crucial cognitive skill in microsurgery that involves comprehending and recalling spatial relationships between objects in space. This skill is fundamental for performing tasks like vessel wall dissection, precise suture placement, and tight knot-tying. The correct placement and spacing of sutures necessitate visuospatial awareness to prevent entangling the suture and catching the back wall of the vessel. In addition, knot-tying and tightening under a microscope are primarily executed using visual perception rather than tactile sensation. *Dexterity* encompasses hand steadiness, the flow of movements, finesse of surgery, and the ability to handle instruments and tissue. Hand steadiness, i.e., the absence of tremors, is essential to handle the micro-instruments comfortably. Dexterous tissue handling is critical to minimize tissue damage, decreasing the risk of vessel thrombosis.

Ghanem et al. [6] examined the outcome of the microsurgical procedures with a particular focus on visuospatial capabilities. They introduced Anastomosis Lapse Index (ALI) to quantify the number of errors made during the process and identified 10 specific errors that could result in anastomotic failures, like thrombosis or leakage. The authors demonstrated that surgeons with more experience had significantly fewer errors and better performance compared to those with less experience. Based on this index, surgeons’ competency levels can be assessed, where a score of greater than 6 indicates a *novice* level, a score between 3 and 6 indicates an *intermediate* level, and a score of less than 3 indicates an *expert* level.

The global rating scale (GRS) is a well-known method for assessing various aspects of hand movements, including *dexterity*. This approach has been shown to be a valid tool with good inter-rater reliability [2, 31]. The GRS uses a 5-point Likert scale to score seven evaluation items, with 1 indicating a failure and 5 representing superior performance. In surgical education, the GRS has been used in various methods to evaluate the skills of surgical trainees in operating rooms, including the objective structured assessment of technical skills (OSATS) [18] and the Ottawa surgical competency operating room evaluation (O-SCORE) [8]. In the context of microsurgery, the GRS inspired the development of the Stanford microsurgery and resident training (SMaRT) scale [30, 34]. The SMaRT scale assesses several essential factors for technical performance, such as instrument handling and tissue handling.

Despite the wide use of the methods mentioned above in the surgeons’ training, the resources and time involved in getting several senior surgeons to observe the overall performance of trainees are the main drawbacks of some of these approaches, e.g., SMaRT. In addition, the observations and judgments coming from this monitoring phase may lead to subjectivity and problems with bias [23]. Moreover, although these approaches give a score for errors during the surgical procedure, most do not elucidate the “tricks” to a successful manipulation, nor do the reasons account for manipulation errors. In other words, these methods lack guidance for trainees to improve their skills in future training. Thus, researchers have shown an ever-growing interest in adopting computer-aided techniques to partially or fully automatize the skill evaluation procedure for optimizing the training and advancement of aspiring microsurgeons into skilled experts [16].

Computer-aided systems, which overcome these limitations and add clarity and consistency to the evaluation process, may offer a promising solution. Considering the existing gap in objective assessment of microsurgical skills, it is imperative to develop a quantitative framework that enables an objective assessment of trainees’ microsurgical anastomosis skills (e.g., similar to [1]). This framework can also serve as a means to provide feedback for skill enhancement. *We hypothesize that it is feasible to differentiate the proficiency levels of microsurgeons, which aligns with the subjective assessment outcomes based on the ALI score.* The central question that drives this study is: how can we effectively create a reliable quantitative evaluation framework that enables accurate assessment and improvement of microsurgical anastomosis skills among surgical trainees? To accomplish this objective, we meticulously choose a collection of well-established metrics from the existing literature on laparoscopic skill assessment, specifically focusing on metrics related to time and instrument kinematics [4, 11, 27]. Furthermore, we draw inspiration from studies on human motor control and hand rehabilitation, incorporating metrics that involve tactile information, such as the force exerted by the fingers on the surgical tools [28].

## Methods and materials

### Subjects

We recruited 12 healthy subjects of different ages ($35.42\pm 9.78$ years), genders (nine males and three females), and handedness (10 right-handed, one left-handed, and one ambidextrous — self-reported) to evaluate the proposed microsurgical assessment framework. All subjects utilized their right hand to handle the needle holder, while their left hand was employed to manipulate the tweezers. Two subjects were robotics students of the École Polytechnique Fédérale de Lausanne (EPFL), Lausanne, Switzerland. The others were surgeons or residents at Geneva University Hospitals (HUG), Geneva, Switzerland. The participants were classified into two distinct groups based on their most recent ALI scores, which served as a benchmark of their current proficiency in microsurgery: novice with six subjects (ALI score $>6$) and intermediate with six subjects ($3\le$ ALI score $\le 6$). The registered ALI scores of subjects were assessed by an expert microsurgeon during the same week of our experimental recording session. Due to apparatus malfunctions, we discarded the data collected from the first novice subject.

### Protocol and task

**Fig. 1:**
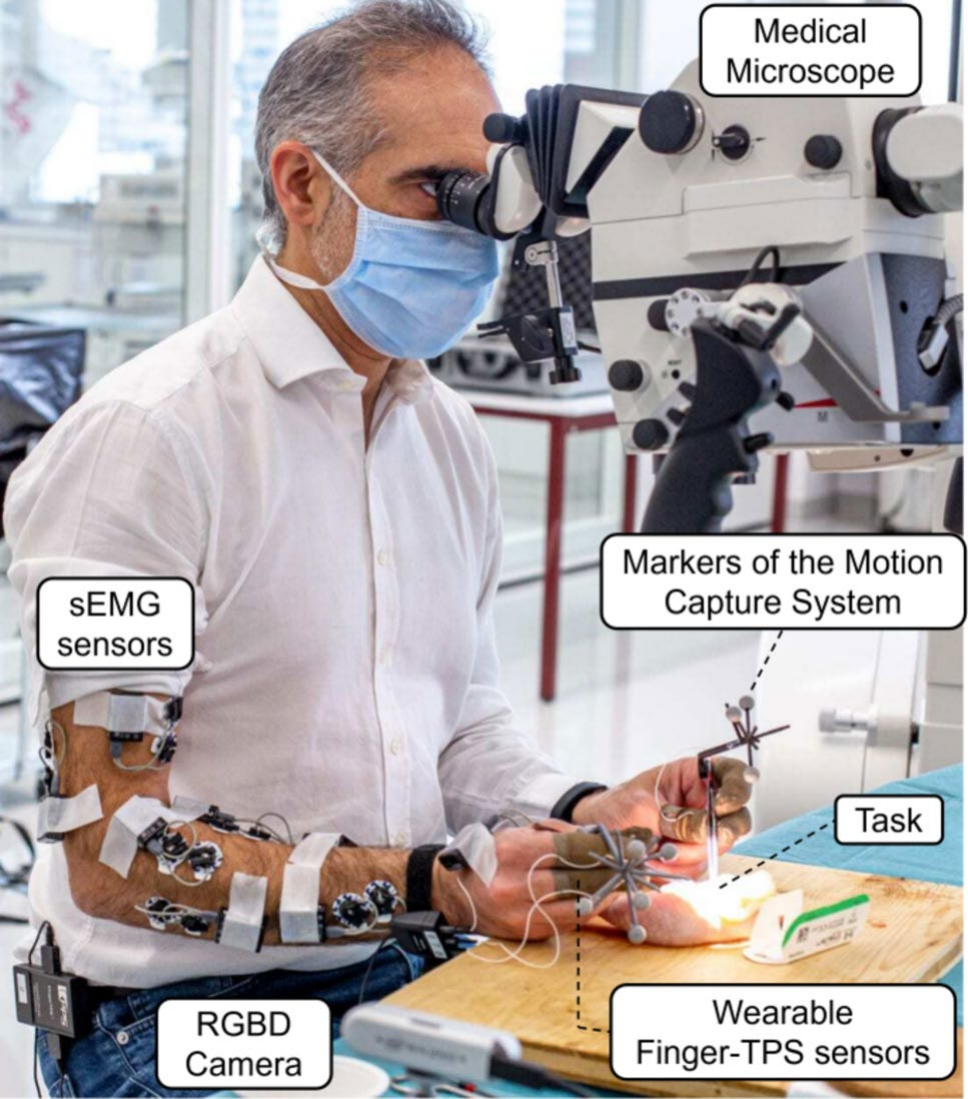
A surgeon performs a microsurgical anastomosis task on a chicken leg while his skill level is being quantitatively evaluated using a set of sensors. An optical motion capture system is employed to track microsurgical instruments. Tactile pressure sensing of fingers (Finger-TPS) is used to extract the finger-generated force values during the task. The task is recorded from different angles using different cameras (including the highlighted RGBD camera) and a medical microscope. Surface electromyographic (sEMG) sensors are mounted on the skin of the forearms to record muscle activities; however, they were not used in the present study (*image courtesy*: www.sfits.ch)

We provided a verbal description of the task, emphasizing the order of the task steps (see below for the task steps), and recording equipment for the subjects prior to the experiments. Subjects gave consent before recording and they signed the SFITS Code of Conduct. The experiment was conducted using ethical practices in accordance with institutional guidelines (the Declaration of Helsinki and reference to the protocol PB_2016-01635, amendment 3 approved by the Commission Cantonal d’Ethique de la Recherche de Geneva). The task was conducted in a surgical workstation, where subjects were instructed to sit on a surgical chair in front of a table, with the surgical microscope and surgical tools placed on the table surface, in front of the subject (see Fig. 1). A variety of practice models, ranging from low-fidelity ones (like silicon tubes) to high-fidelity alternatives (for instance, live animals), are extensively employed in microsurgical training. For our investigation, we selected the chicken leg model, an intermediate-fidelity option, due to its advantageous attributes of availability, reproducibility, cost-effectiveness, and ethical acceptability [10]. This model, however, does not capture all difficulties faced when handling living tissues. The study participants were assigned to conduct a surgical anastomosis procedure on the chicken leg model. This procedure involved making an incision in a blood vessel of the chicken leg and subsequently suturing the severed ends together, utilizing the end-to-end surgical anastomosis technique.

**Fig. 2:**
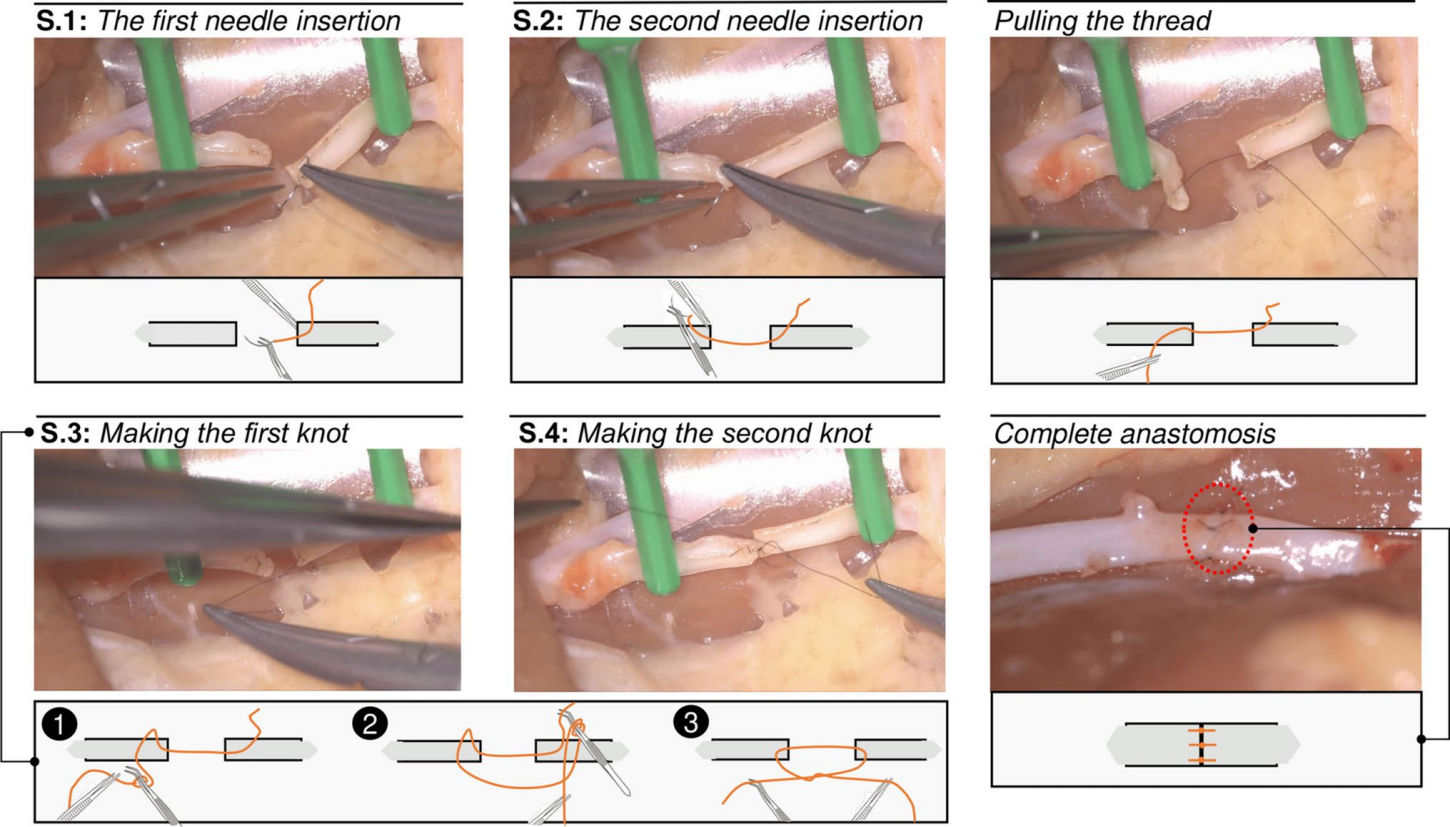
The photo snapshots shown in the figure represent the task sequence (task segments). These are taken from one of the recorded videos during the task performance by a surgeon. A conceptual illustration of each segment is also provided, except for the fifth segment (identical to the fourth segment). Three crucial moments in making a knot are displayed in the fourth segment (in the highlighted gray box)

In a standard anastomosis, multiple stitches are necessary to connect the separated structures, and these stitches are typically not of equal difficulty due to varying constraints. For the first stitch, the main challenge is to ensure that the two ends are correctly positioned in the field. When performing additional stitches, more constraints arise as more vessel parts are connected, resulting in reduced access space for the tools. Quality criteria outlined in the ALI score, such as equal distance between knots and continuity of the anastomosis line, must be considered when working with previous knots. Finally, the last stitches performed on the back side of the vessel are also challenging, as the vessel must be flipped over to allow for access. To ensure consistency in the task’s difficulty level across all subjects and trials, we asked each subject to repeat the first stitch only. By employing this approach, we ensured a fair comparison by only evaluating stitches of similar difficulty levels, thereby avoiding the comparison of stitches with varying levels of complexity. The subjects utilized a pair of microsurgical forceps (thumb-driven tweezers), a needle driver (needle holder), and a microscope to perform the task. The subjects manipulated the tweezers and a needle holder using their non-dominant (left) and dominant (right) hands, respectively. A detailed sequence of the task is illustrated in Fig. 2.

To perform a stitch, the subjects used the needle holder to grab the needle from the micro-suture, which consisted of a small needle and an attached thread. They then inserted the needle tip into an entry point on one vessel while holding the vessel with the forceps, passing the needle through the vessel wall (Fig. 2-S.1). Next, the subjects passed the needle through the wall of the second piece of the vessel, entering the lumen of the artery and exiting the vessel wall (Fig. 2-S.2). Using the needle driver, the subjects pulled the thread through, leaving a small end to allow for tying knots. They adjusted the amount of thread they needed to complete the knots (i.e., pulling the thread). They formed a thread loop around the needle holder using the forceps to make the first knot. The needle holder caught the tail of the thread within the loop, and both hands pulled each side of the thread to tighten the knot (Fig. 2-S.3). The subjects then repeated the same manipulation procedure to make the second knot (Fig. 2-S.4). Finally, they used scissors to cut the thread on both sides of the knot. Each participant performed this task three times, completing three trials in total.

### Measurement system

The recorded data, including (1) time, (2) visual information obtained from a set of cameras and a medical microscope, (3) kinematics estimated by a portable OptiTrack system (Natural Point Inc., USA) with an estimation frequency of 120*.*0 Hz, and (4) exerted force information measured by the Finger-TPS system (Medical Tactile Inc., USA) with an update frequency of 40*.*0 Hz, are analyzed offline to calculate the metrics introduced in Table 1. Segmentation of the videos to extract the anastomosis task segments, which are most relevant to identify skill proficiency, was performed manually.

**Table 1:** Our proposed objective skill assessment framework. We use $|\cdot |$ to represent the absolute value of a scalar and $\overline{\cdot }$ to indicate the mean value of a signal. The *p*-norm, ${\mathcal{L}}^{p}$-norm, of a vector ${\varvec{x}}={\left[{x}_{1},{x}_{2},\dots ,{x}_{n}\right]}^{T}$ in the *n-*dimensional real-vector space ${R}^{n}$ is used which is defined as ${\Vert {\varvec{x}}\Vert }_{p}={\left({\sum }_{i=1}^{n}{\left|{x}_{i}\right|}^{p}\right)}^\frac{1}{p}$. The Savitzky-Golay filter (polynomial order $=3$ and window size $=5$) is used to obtain the time derivatives of each signal, e.g., ${\varvec{v}}(t)$,  ${\varvec{a}}({t})$, and ${\dot{f}}_{i}\left(t\right)$

Symbol	Name	Explanation	Definition
*Time*			
$\mathcal{M}_{\Delta T}$	Completion time [s]	The amount of time required to complete a task segment	${\mathcal{M}}_{\Delta T}=\left({k}_{f}-{k}_{0}\right)\times \mathrm{sampling\, period}$
${\mathcal{M}}_{{{\text{T}}}_{{\text{idle}}}}$	Idle time [s]	The time duration in which the instrument is held motionless, i.e., the tool’s velocity is lower than a predefined threshold ${v}^{\star }$	${{\mathcal{M}}_{T}}_{{\text{idle}}}=\mathrm{time\, duration\, where}$ $\left|\left|{\varvec{v}}\left({k}\right)\right|\right|_{2}\le {{v}}^{\star }$
*Motions of the instruments (kinematics)*			
$\mathcal{\mathcal{M}}_{{\text{PL}}}$	Path length [cm]	The total path traversed by the tool tip of the instrument in the 3-dimensional Cartesian coordinates	${\mathcal{M}}_{{\text{PL}}}={\sum }_{{k=k}_{0}}^{{k}_{f}}{\Vert {\varvec{v}}\left(k\right)\Vert }_{2}$
${\mathcal{M}}_{{\text{MS}}}$	Motion smoothness (log dimensionless jerk)	It is defined based on the instantaneous jerk vector ${\varvec{j}}(t)$, i.e., the time derivative of the acceleration ${\varvec{a}}(t)$ vector	${\mathcal{M}}_{{\text{MS}}}=-{\text{ln}}\left(\frac{{{M}}_{\Delta T}^{3}}{{{\varvec{v}}}_{\mathbf{m}\mathbf{a}\mathbf{x}}^{2}} {\sum }_{k={k}_{0}}^{{k}_{f}}{\Vert {\varvec{j}}(k)\Vert }_{2}^{2}\right)$
$\mathcal{\mathcal{M}}_{{\text{EoV}}}$	Economy of volume [%]	The relation between the maximum covered volume and path length	${\mathcal{M}}_{{\text{EoV}}}=\frac{\sqrt[3]{{{M}}_{{\text{MV}}}}}{{{M}}_{{\text{PL}}}}\times 100$ where ${\mathcal{M}}_{{\text{MV}}}={\prod }_{j}|{\text{max}}\left({p}_{j}\right)-{\text{min}}({p}_{j})|$
${\mathcal{M}}_{{\text{BD}}}$	Bimanual dexterity	It specifies the correlation between the tool tips’ velocity, controlled by the left and right hands. It serves as a coordination metric between the two hands of the surgeons	${\mathcal{M}}_{{\text{BD}}}=\frac{\sum_{k={k}_{0}}^{{k}_{f}}\left({v}_{l}(k)-{\overline{v} }_{l}\right)\left({v}_{r}(k)-{\overline{v} }_{r}\right)}{\sum_{k={k}_{0}}^{{k}_{f}}{\left({v}_{l}(k)-{\overline{v} }_{l}\right)}^{2}\sum_{k={k}_{0}}^{{k}_{f}}{\left({v}_{r}(k)-{\overline{v} }_{r}\right)}^{2}}$
*Tactile pressure sensing of fingers*			
${\mathcal{M}}_{{\text{NoP}}}$	Number of presses	The number of estimated pinching actions on the tools to close the instruments (the needle holder and tweezers)	${\mathcal{M}}_{{\text{NoP}}}=\mathrm{number\,of\,local\,maxima\,in\,}\mathcal{A}\left(t\right)$

We carefully designed our measurement system to minimize the cognitive and physical workloads on surgeons during tasks. For instance, we employ lightweight 3D-printed structures for the OptiTrack markers to minimize any alteration in the weight and manipulability of surgical instruments. Additionally, the Finger-TPS wearable sensors we use are lightweight and securely affixed to the surgeons’ fingers. In terms of sensor calibration, we ensure bias reduction by individually calibrating the Finger-TPS sensors for each subject before every experiment, adhering to the software and procedures recommended by Medical Tactile Inc. The same calibration procedures are followed for the OptiTrack system.

### Quantitative metrics

The quantitative metrics utilized in our study are presented in Table 1 and are categorized into three groups: time metrics, instrument motion metrics, and finger force metrics. These metrics are carefully selected after a thorough review of existing literature in the surgery skill assessment (or similar fields), considering (i) time metrics [3, 4, 11, 25, 26] to track the task completion and idle time; (ii) tools’ motions [4, 5, 7, 9, 11, 12, 21, 22, 25–27, 32, 33] to monitor to track the traveled path, jerky motions, economy of movements, and dexterity; and (iii) tactile pressure sensing of fingers to measure the applied force on the tools to obtain the amount of pinch/release actions on them [28]. We hypothesize that as surgeons advance in their surgical expertise, there will be an enhancement in the reported values of the chosen quantitative metrics, aligning with factors assessed in subjective evaluation tools such as SMaRT (e.g., operational flow can be quantified by the idle time metric; instrument handling by path length and smoothness metrics).

We define two temporal metrics: task completion (${\mathcal{M}}_{\Delta T}$) and task idle time (${{\mathcal{M}}_{T}}_{{\text{idle}}}$). The former measures the time when the studied tool(s) are still, i.e., the velocity’s ${L}_{2}$-norm of its tip is less than a predefined threshold. The kinematics-related (instruments’ motions) metrics are calculated based on the position ${{\varvec{p}}}_{i}=\left[{p}_{x}, {p}_{y}, {p}_{z}\right]\in {R}^{3}$ and orientation ${{\varvec{\epsilon}}}_{i}=\left[{\epsilon }_{x}, {\epsilon }_{y}, {\epsilon }_{z}\right]\in {R}^{3}$ vectors of the employed instruments’ tips, where $i\in \left\{l,r\right\}$ represent left (the tweezers) and right (the needle holder) instruments, respectively. The linear velocity and acceleration vectors are represented as ${\varvec{v}}(t)\in {R}^{3}$ and ${\varvec{a}}(t)\in {R}^{3}$, respectively. Regarding the Finger-TPS measurement, the normalized derivative of the raw force signal was used for comparison requirements. The normalization procedure was carried out concerning the signal’s maximum value. We define the “activity signal” of each hand as follows: ${\mathcal{A}}_{j}\left(t\right)={\Sigma }_{i}\,{\dot{f}}_{i}(t)$, where $i\in \{$ thumb, middle, and index fingers} and $j\in${left and right hands}, which is an indicator of the activities followed by the operator during the task (e.g., pinching and releasing). This indicates the sum of the derivatives of the force signals measured on active fingers. This definition is used to calculate the ${\mathcal{M}}_{{\text{NoP}}}$ metric.

### Statistical analysis

To effectively illustrate the results for the metrics in each segment, box plots are used. The red “$+$” signs and filled green triangles indicate outliers and average values of each distribution, respectively. This means that any extreme data points that significantly deviate from the overall pattern are considered outliers and excluded from the analysis (based on the quartiles). In addition, we employ the Shapiro–Wilk test to assess the normality of each distribution. If a distribution passes the normality test, we denote it with an “*” sign alongside the corresponding group name (e.g., N* for the novice group and I* for the intermediate group). Moreover, the median (*M*) and the interquartile range (IQR) values are denoted by *M* (IQR) pair in the text. For the statistical hypothesis testing, we employ different approaches based on the distribution characteristics. Specifically, if we have two normally distributed sample groups, we utilize the *t*-test to assess the statistical significance. However, if one or both distributions do not meet the criteria for normality, we opt for the Wilcoxon test as an alternative (both performed with a two-sided approach). The corresponding *p*-values are reported in the text. In the event of a significant difference, we respectively represent it in the plots using the symbols “${T}^{\star }$” and “${W}^{\star }$” placed over a horizontal line. In this study, we report statistically significant differences at the significance level of $\alpha =.05$. For both tests, the degree of freedom (df) is calculated by (${n}_{N}+{n}_{I}-2$) where ${n}_{N}$ and ${n}_{I}$ are the sample size of novice and intermediate groups, respectively. These two parameters are presented in the plots.

## Data analysis

The task for each subject is analyzed within four distinct segments (see Fig. 2), with each segment being a discrete signal sequence that starts at the initial sample ${k}_{0}$ and ends at the final sample ${k}_{f}$ (see the “Protocol and task” section). The metrics we use are calculated based on the data within each sequence. The descriptive statistics for the employed metrics are presented in Table 2, showcasing the key findings and numerical summaries.

**Table 2:** The descriptive statistics of the employed metrics. The results are reported using the median (*M*) and the interquartile range (IQR) values, denoted by *M* (IQR) pair. The entries presented in italics signify that there is no statistically significant difference between the groups at the 0.05 significance level

Metric [quantity]	Employed instrument	Segment 1 (S.1)		Segment 2 (S.2)		Segment 3 (S.3)		Segment 4 (S.4)	
		*Novice*	*Intermediate*	*Novice*	*Intermediate*	*Novice*	*Intermediate*	*Novice*	*Intermediate*
${\mathcal{M}}_{\Delta T}\,[s]$	Both	$39.92\,(29.11)$	$19.11\,(15.29)$	$26.03\,(12.88)$	$11.21\,(12.81)$	$25.81\,(9.84)$	$10.05\,(4.81)$	*11.64 (5.90)*	*5.75 (5.28)*
${\mathcal{M}}_{\text{T}_{idle}}\,[s]$	Tweezers	*10.49 (7.31)*	*4.68 (10.52)*	$7.45\,(19.53)$	$2.33\,(2.80)$	$7.15\,(7.22)$	$1.39\,(2.44)$	$1.92\,(4.95)$	$0.53\,(0.68)$
	Needle holder	$15.17\,(13.65)$	$7.75\,(12.34)$	$10.13\,(17.55)$	$4.61\,(12.05)$	$11.62\,(7.13)$	$2.26\,(2.73)$	$3.30\,(4.48)$	$0.91\,(0.64)$
	Both	*8.21 (3.32)*	* 3.19 (9.65)*	$4.86\,(17.87)$	$1.30\,(2.09)$	$5.39\,(2.93)$	$1.02\,(1.78)$	$1.31\,(3.61)$	$0.28\,(0.31)$
${\mathcal{M}}_{{\text{PL}}}\,[cm]$	Tweezers	$36.18\,(15.62)$	$15.02\,(12.26)$	$17.73\,(13.75)$	$8.62\,(7.60)$	$41.91\,(38.45)$	$14.75\,(16.35)$	$23.78\,(18.46)$	$6.78\,(4.60)$
	Needle holder	$43.50\,(37.94)$	$17.93\,(16.87)$	$19.30\,(16.44)$	$9.49\,(6.65)$	$35.74\,(33.10)$	$13.50\,(13.93)$	$15.43\,(14.93)$	$4.93\,(2.71)$
${\mathcal{M}}_{{\text{MS}}}\,[-]$	Tweezers	$-27.82\,(2.08)$	$-25.31\,(1.64)$	$-27.05\,(2.75)$	$-23.79\,(2.48)$	$-26.62\,(0.81)$	$-22.86\,(1.66)$	$-24.24\,(3.10)$	$-21.02\,(4.10)$
	Needle holder	$-27.94\,(3.08)$	$-24.62\,(2.04)$	$-26.39\,(3.31)$	$-23.18\,(2.30)$	$-26.37\,(1.32)$	$-23.28\,(2.59)$	$-23.56\,(1.93)$	$-21.18\,(3.95)$
${\mathcal{M}}_{{\text{EoV}}}\,[\%]$	Tweezers	$3.65\,(3.19)$	$5.68\,(2.79)$	$4.1\,(2.04)$	$6.79\,(4.76)$	$5.90\,(2.21)$	$12.66\,(7.04)$	$9.30\,(6.85)$	$15.47\,(11.65)$
	Needle holder	$6.16\,(3.22)$	$11.59\,(3.71)$	$7.07\,(3.37)$	$10.51\,(2.77)$	$8.50\,(4.07)$	$17.30\,(12.03)$	$9.31\,(6.29)$	$18.69\,(12.47)$
${\mathcal{M}}_{{\text{BD}}}\,[-]$	Both	$0.57\,(0.10)$	$0.66\,(0.09)$	*0.62 (0.17)*	*0.57 (0.14) *	*0.67 (0.20)*	*0.74 (0.14)*	*0.63 (0.21)*	*0.70 (0.17)*
${\mathcal{M}}_{{\text{NoP}}}\,[\#]$	Left hand	$20.00\,(9.50)$	$8.00\,(3.00)$	*8.50 (12.75)*	*8.50 (8.50)*	*5.50 (3.50)*	*6.00 (2.25)*	*5.00 (3.25)*	*4.00 (1.25)*
	Right hand	$13.00\,(14.50)$	$6.50\,(3.75)$	$10.00\,(8.00)$	$4.00\,(2.00)$	$9.00\,(17.50)$	$4.00\,(6.00)$	*5.00 (5.00)*	*4.50 (4.00)*

### Metrics of time

**Fig. 3:**
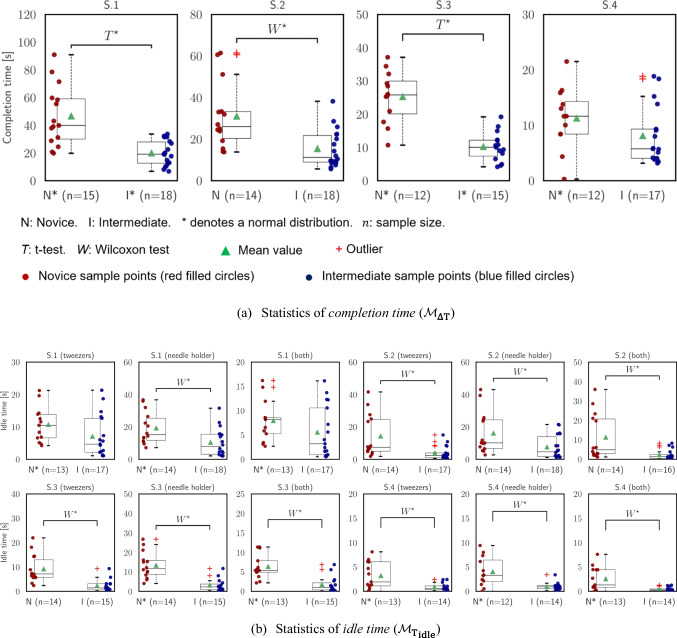
Statistical analysis of time-related metrics. The legend applied in subfigure **a** also applies to the subsequent figures. **a** Statistics of *completion time* (${\mathcal{M}}_{\mathrm{\Delta T}}$). **b** Statistics of *idle time* (${{\mathcal{M}}_{{\text{T}}}}_{{\text{idle}}}$)

The results of time-related metrics are presented in Table 2 and Fig. 3. The intermediate subject group demonstrated faster execution times across all segments compared to the novice subjects (see Fig. 3a). Statistically significant differences were observed for the first three segments (S.1: independent *t*-test, $t=4.76, p<.001$; S.2: Wilcoxon rank-sum, $U=3.15, p =.002$; S.3: independent *t*-test, $t =6.33, p<.001$). Furthermore, we observe improvements from the first segment to the second one and from the third segment to the fourth one, indicating enhanced performance in repetition of the same segment [S.1 to S.2: $39.92 \, (29.11)$ to $26.02\,(12.88)$ for novices, and $19.11\,(15.29)$ to $11.21\,(12.81)$ for intermediates; S.3 to S.4: $25.81\,(9.84)$ to $11.64\,(5.90)$ for novices, and $10.05\,(4.81)$ to $5.75\,(5.28)$ for intermediates].

Intermediate participants exhibited shorter idle times when manipulating the needle holder, the tweezers, or both, compared to their novice counterparts (see Fig. 3b). The findings indicate that participants at an intermediate level showed greater efficiency in utilizing their idle time during these tasks. Moreover, notable enhancements were observed when participants repeated the first-to-second and third-to-fourth segments, suggesting a learning effect or increased proficiency over time. This improvement led to better performance during subsequent repetitions of the tasks. However, it is worth noting that the statistical significance was not evident in the first segment when the tweezers and both instruments were considered [S.1 (needle holder): Wilcoxon rank-sum, *U* = 2.47, *p* = .01; S.2 (tweezers): Wilcoxon rank-sum, *U* = 3.06, *p* = .002; S.2 (needle holder): Wilcoxon rank-sum, *U =* 2.47, *p* = .01; S.2 (both): Wilcoxon rank-sum, *U* = 2.97, *p* = .003; S.3 (tweezers): Wilcoxon rank-sum, *U* = 3.84, *p* < .001; S.3 (needle holder): Wilcoxon rank-sum, *U* = 4.05, *p* < .001; S.3 (both): Wilcoxon rank-sum, *U* = 3.57, *p* < .001; S.4 (tweezers): Wilcoxon rank-sum, *U* = 2.23, *p* = .02; S.4 (needle holder): Wilcoxon rank-sum, *U* = 2.78, *p* = .005; S.4 (both): Wilcoxon rank-sum, *U* = 2.38, *p* = .02].

### Metrics of instruments’ motions

**Fig. 4:**
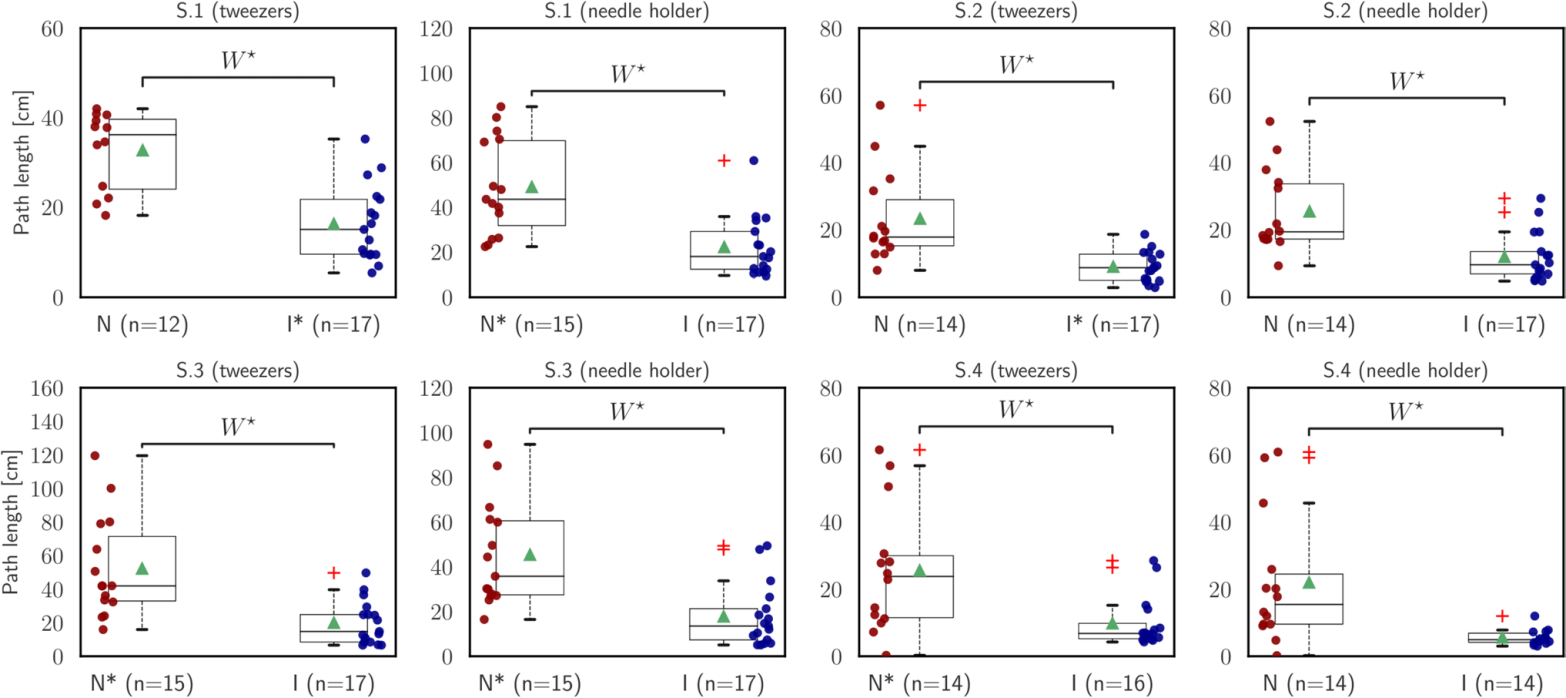
Statistical analysis of *path length* (${\mathcal{M}}_{{\text{PL}}}$)

**Fig. 5:**
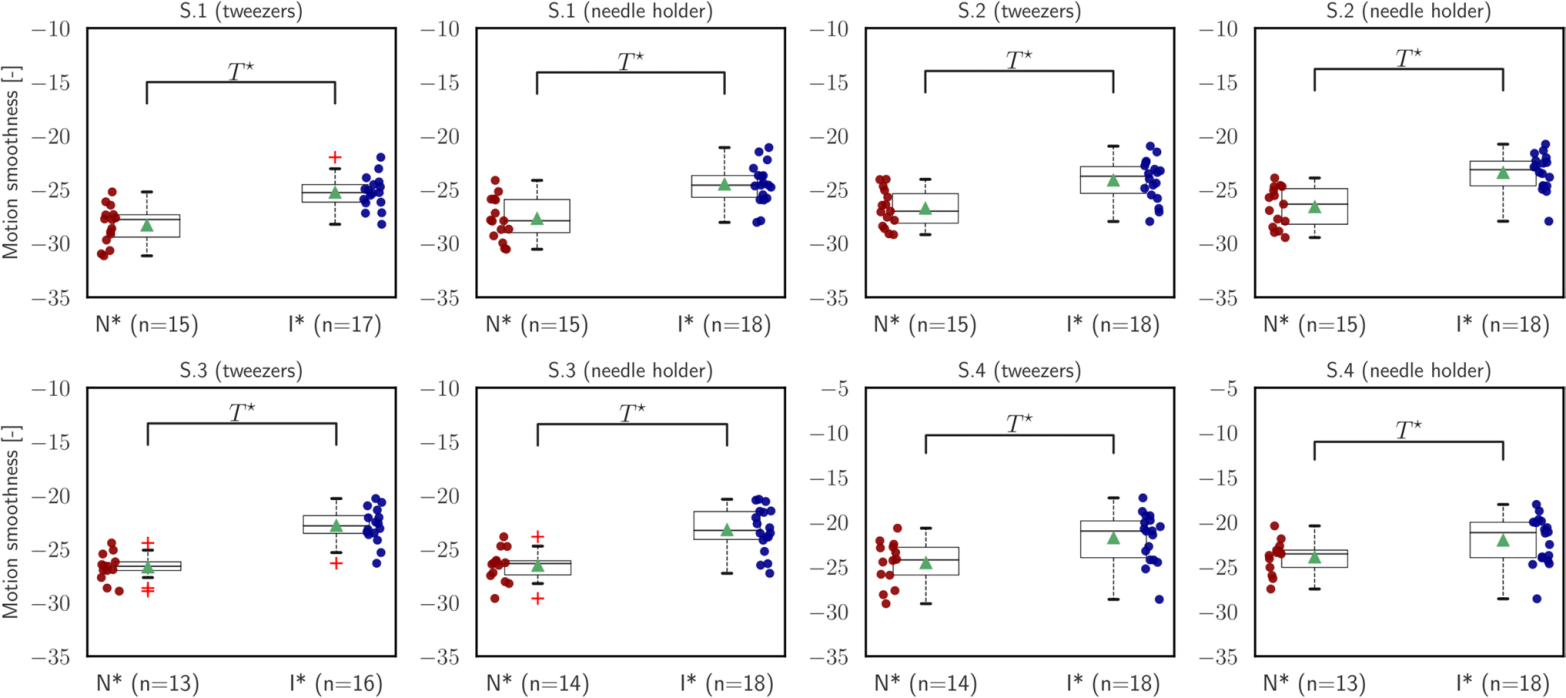
Statistical analysis of *motion smoothness* (${\mathcal{M}}_{{\text{MS}}}$)

**Fig. 6:**
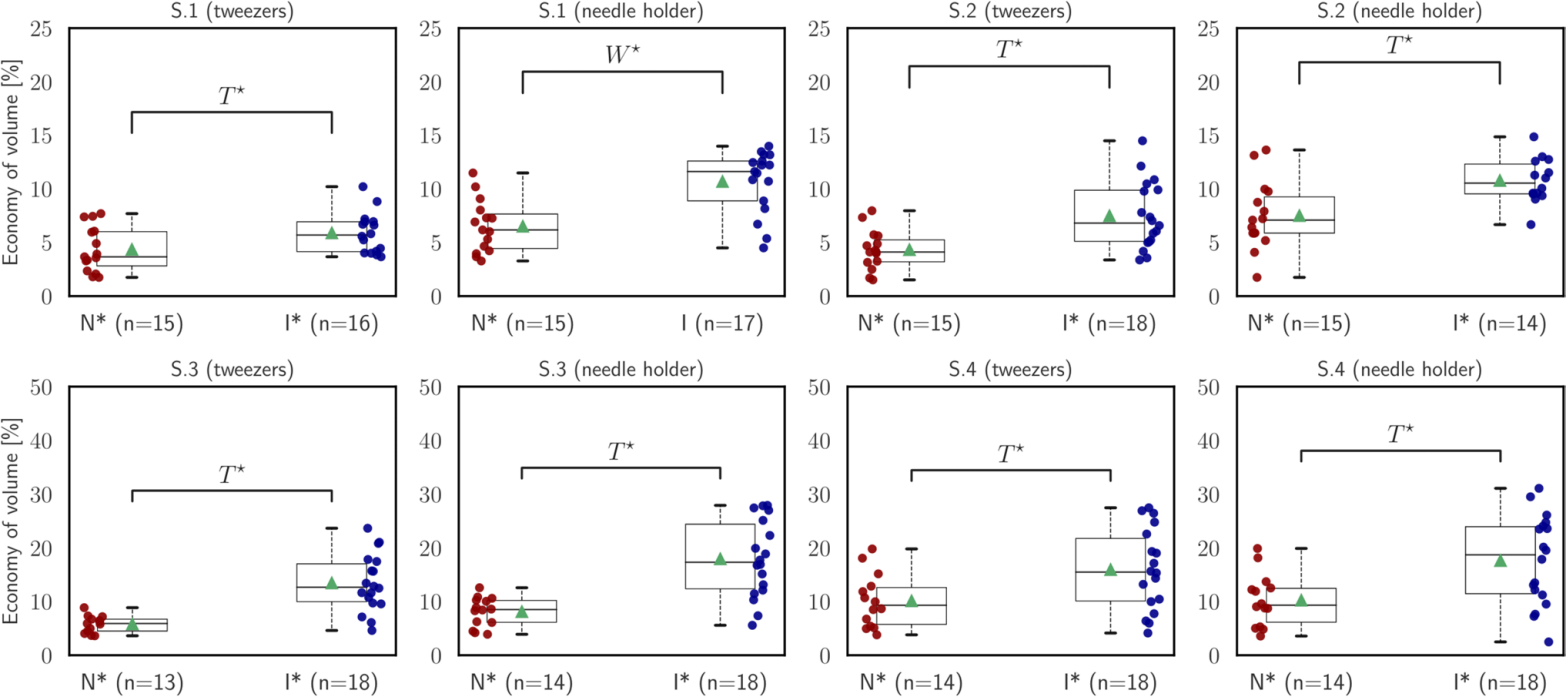
Statistical analysis of *economy of volume* (${\mathcal{M}}_{{\text{EoV}}}$)

**Fig. 7:**
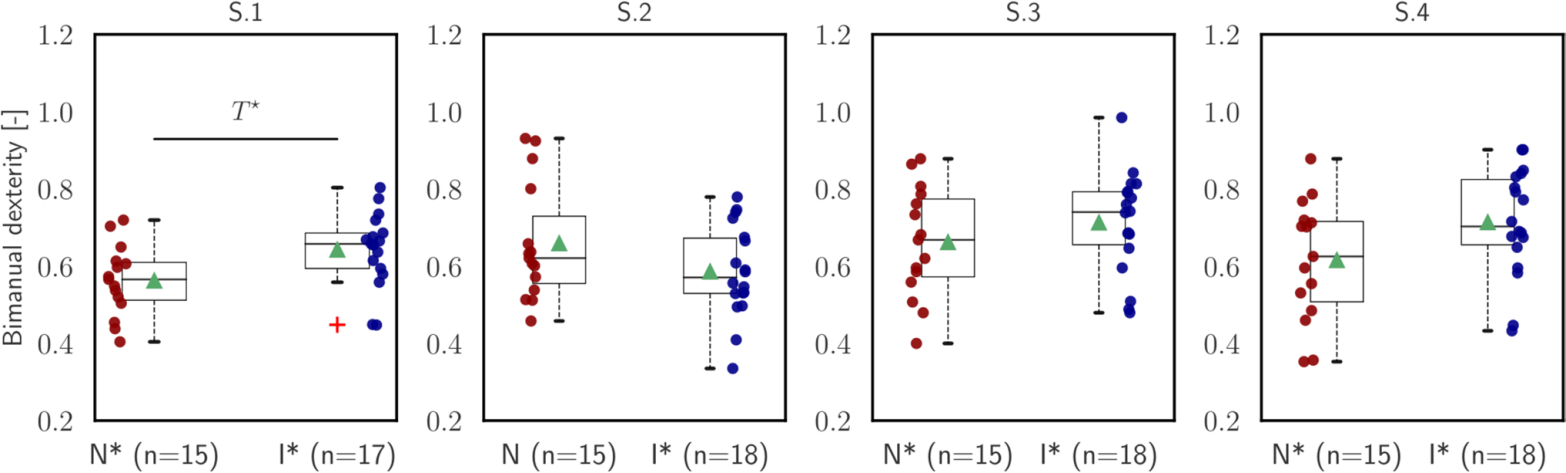
Statistical analysis of *bimanual dexterity* (${\mathcal{M}}_{{\text{BD}}}$)

The results of the metrics related to the instruments’ motions are presented in Table 2 and Figs. 4, 5, 6, and 7.

The initial measure is the path length (${\mathcal{M}}_{{\text{PL}}}$) expressed in centimeters [cm]. This metric quantifies the total distance covered by the instrument’s tool tip in three-dimensional Cartesian coordinates. It offers valuable information about the overall range of movement exhibited by the instrument during each segment of the task. In all segments and for both instruments, the intermediate group of participants demonstrated significantly shorter path lengths compared to the novices [S.1 (tweezers): Wilcoxon rank-sum, $U=3.63, p<.001$; (needle holder): Wilcoxon rank-sum, $U=3.72, p<.001$; S.2 (tweezers): Wilcoxon rank-sum, $U=3.81, p<.001$; S.2 (needle holder): Wilcoxon rank-sum, $U=3.25, p=.001$; S.3 (tweezers): Wilcoxon rank-sum, $U=3.53, p<.001$; S.3 (needle holder): Wilcoxon rank-sum, $U=3.68, p<.001$; S.4 (tweezers): Wilcoxon rank-sum, $U=2.87, p=.004$; S.4 (needle holder): Wilcoxon rank-sum, $U=3.40, p<.001$]. These statistically significant differences underscore the intermediates’ capacity to execute more accurate and efficient movements (Fig. 4).

The second measure is referred to as motion smoothness (${\mathcal{M}}_{{\text{MS}}}$), and it is determined by examining the instantaneous jerk vector ${\varvec{j}}(t)$. This vector corresponds to the time derivative of the acceleration vector ${\varvec{a}}(t)$. Motion smoothness, without specifying a quantity, offers an evaluation of how smoothly and consistently the instrument moves, by capturing the rate at which acceleration changes over time. The outcomes shown in Fig. 5 demonstrate that intermediate participants exhibited smoother trajectories in all segments for both instruments. These disparities in motion smoothness were determined to be statistically significant throughout the entire analysis, indicating that intermediates displayed more fluid and stable movements with the instruments compared to novices [S.1 (tweezers): independent *t*-test, $t=-5.20, p<.001$; S.1 (needle holder): independent *t*-test, $t=-4.74, p<.001$; S.2 (tweezers): independent *t*-test, $t=-4.05, p<.001$; S.2 (needle holder): independent *t*-test, $t=-5.10, p<.001$; S.3 (tweezers): independent *t*-test, $t=-6.97, p<.001$; S.3 (needle holder): independent *t*-test, $t=-4.96, p<.001$; S.4 (tweezers): independent *t*-test, $t=-2.93, p=.006$; S.4 (needle holder): independent *t*-test, $t=-2.16, p=.04$].

The third measure, called the economy of volume (${\mathcal{M}}_{{\text{EoV}}}$), is expressed as a percentage and assesses the relationship between the maximum volume covered and the path length. It quantifies how efficiently the instrument covers the volume in relation to the distance traveled. A higher percentage of the economy of volume indicates that the instrument covers a larger volume while taking a shorter path, reflecting a more efficient use of motion. Based on the findings depicted in Fig. 6, intermediate participants demonstrated more economical performance in completing all segments compared to the novices, using both instruments. These disparities in the economy of volume were statistically significant in all instances [S.1 (tweezers): independent *t*-test, $t=-2.15, p=.04$; S.1 (needle holder): Wilcoxon rank-sum, $U=-0.45, p<.001$; S.2 (tweezers): independent *t*-test, $t=-3.43, p=.002$; S.2 (needle holder): independent *t*-test, $t=-3.25, p=.003$; S.3 (tweezers): independent *t*-test, $t=-5.03, p<.001$; S.3 (needle holder): independent t-test, $t=-4.84, p<.001$; S.4 (tweezers): independent *t*-test, $t=2.48, p=.02$; S.4 (needle holder): independent *t*-test, $t=-2.94, p=.006$]. This indicates that intermediates achieved a higher level of efficiency in covering the required volume relative to the distance traveled, highlighting their enhanced instrument control and precision.

The fourth measure, referred to as bimanual dexterity (${\mathcal{M}}_{{\text{BD}}}$), evaluates the correlation between the speed of the tool tips controlled by the left and right hands. It serves as a coordination metric, indicating the degree of synchronization and dexterity between the two hands of the surgeons. By examining the correlation in tool tip speeds, the bimanual dexterity metric provides insights into the level of coordination achieved during the surgical task. The outcomes are depicted in Fig. 7. In all segments except the second one, intermediate participants demonstrated more coordinated movements compared to the novices. However, statistically significant differences were only observed in the first segment (S.1: independent *t*-test, $t=2.39, p=.02$). This suggests that intermediate participants exhibited higher levels of bimanual dexterity and coordination, particularly in the initial stage of the task.

### Metric of fingers’ force

**Fig. 8:**
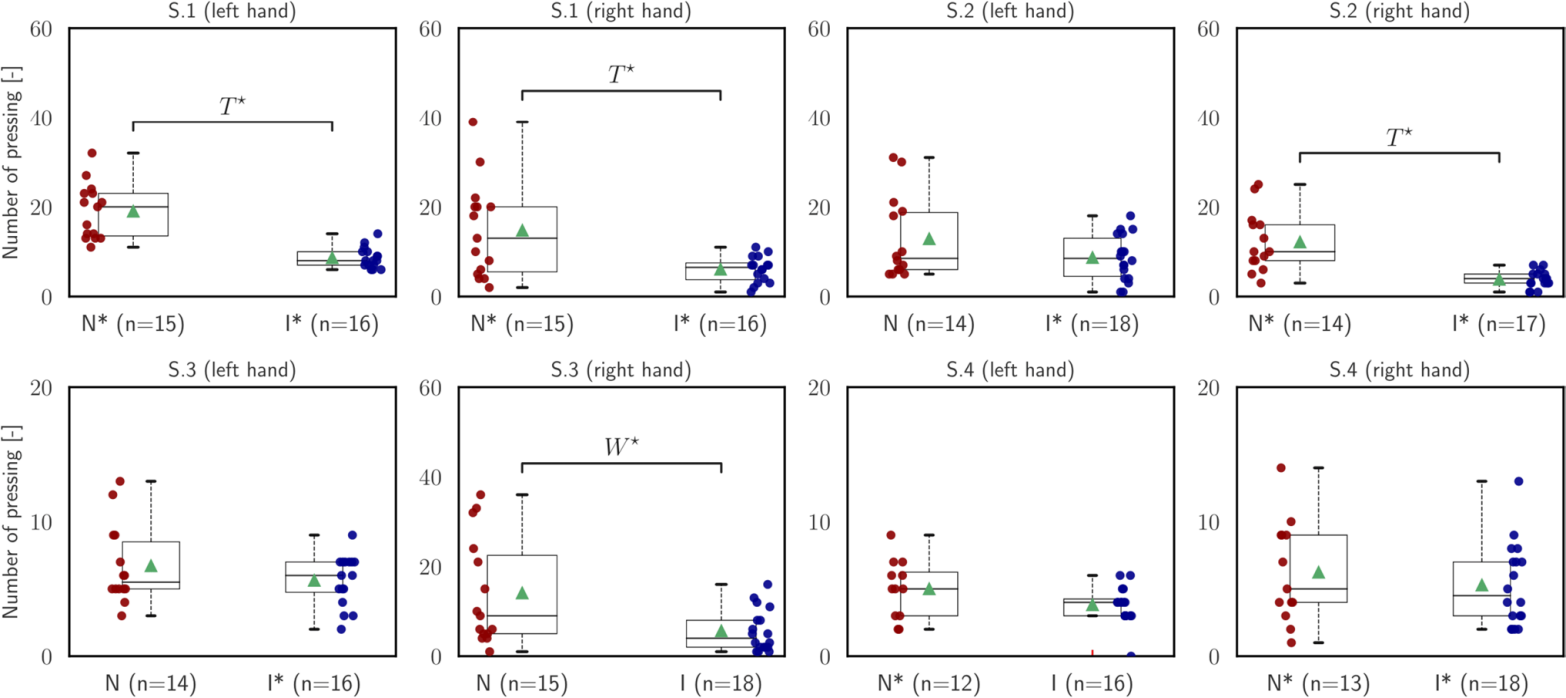
Statistical analysis of *number of presses* (${\mathcal{M}}_{{\text{NoP}}}$)

The results of the force analysis conducted on the subjects’ fingers for the suggested metric, namely the number of presses (${\mathcal{M}}_{{\text{NoP}}}$), are presented in Table 2 and Fig. 8. This metric quantifies the estimated number of pinching actions performed on the instruments, involving the closure of the needle holder manipulated by the right hand and the tweezers manipulated by the left hand. The Finger-TPS sensors mentioned earlier were utilized to track the applied force on the thumb, index, and middle fingers of each hand during instrument manipulation. Intermediate participants applied fewer presses on both instruments compared to the novices (see Fig. 8). The differences were statistically significant in the first segment for both hands, in the second segment for the right hand, and in the third segment for the right hand as well [S.1 (left hand): independent *t*-test, $t=6.29, p<.001$; S.1 (right hand): independent *t*-test, $t=3.12, p=.004$; S.2 (right hand): independent *t*-test, $t=4.90, p<.001$; S.3 (right hand): independent *t*-test, $t= 2.26, p=.02]$.

## Discussion

The objective of this study was to quantitatively evaluate the psychomotor skills of surgeons performing microsurgical anastomosis operations. This was achieved by identifying a comprehensive set of key performance metrics (refer to Table 1). The study involved measuring multiple variables, such as the time taken to complete the task, the kinematics of the instruments, and the force exerted by the fingers on the instruments. As a result, we were able to differentiate the proficiency levels of the participant groups. The intermediates consistently outperformed the novices in most of task segments, indicating their superior performance and skill set. These results align with our initial hypotheses and establish a distinct disparity in skill levels among the participants.

The analysis of the metrics used in this study reveals a consistent pattern across the entire surgical task and participants, showing that intermediates generally outperform novices in the given task. However, there is an exception in the second segment regarding bimanual dexterity, where novices demonstrate slightly superior performance. It is worth noting that this improvement in performance can be attributed to the simultaneous movement of both hands without necessarily enhancing the overall outcome of the procedure. Thus, caution should be exercised when assessing psychomotor skills solely based on this metric.

Significant differences are observed in the path length (${\mathcal{M}}_{{\text{PL}}}$), motion smoothness (${\mathcal{M}}_{{\text{MS}}}$), and economy of volume (${\mathcal{M}}_{{\text{EoV}}}$) metrics across all segments, indicating notable variations in performance between participants. However, it is important to acknowledge that not all the differences in the other metrics are statistically significant across all segments. This can be attributed to the limited sample data available for analysis. With a larger sample size and more subjects, it is likely that significant differences would be observed in all segments for these metrics as well.

Moreover, the findings of this study, particularly in relation to time-related metrics and path length, emphasize the inclination of novices to apply the techniques they have acquired during their studies and residency. This is evident through longer task completion times (${\mathcal{M}}_{\Delta T}$), increased idle time (${{\mathcal{M}}_{T}}_{{\text{idle}}}$), and greater generated path lengths (${\mathcal{M}}_{{\text{PL}}}$). These results indicate that novices may still be in the process of refining their skills and implementing the learned techniques, which can affect their overall performance in terms of efficiency and precision. In line with similar studies in the context of surgery such as Hofstad’s research [11] and Ebina’s research [4], our findings regarding time and kinematics analysis support these observations.

Additionally, our study introduced a novel assessment of the number of fingers’ presses on the tools, which quantifies the frequency of pinching and releasing actions during the task. The results demonstrate that intermediates exhibit fewer finger presses compared to novices. Significantly different patterns were observed in the first segment for both hands, the right hand in the second segment, and the right hand in the third segment. These findings shed light on the distinctive patterns of instrument manipulation displayed by intermediates and novices, highlighting the importance of considering such metrics when evaluating skill levels.

This study is subject to several limitations that should be acknowledged. To begin with, the identification of task segments relied on manually reviewing recorded videos. This may however be subject to potential errors or subjective judgments. To mitigate this limitation, automating the segmentation process would be beneficial. Automating the entire process from segmentation to extracting metrics for skill assessment may lead to more effective means to assess skill proficiency. It would further allow for automatic identification of the parts of the tasks where subjects perform less well, as well as reducing subjectivity in data analysis. Overall, this would enhance the accuracy and objectivity of the study’s findings.

This study used the ALI score as the sole metric to assess surgical skills. Conducting cross-validation studies with other established assessment methods such as the Surgical Task Assessment Rating Tool (SMaRT) would be advantageous to further assess the accuracy and reliability of the proposed metric for quantifiable skill assessment.

The metrics considered here provide only a partial assessment of skills. The integration of additional metrics, such as physiological signals like surface electromyography (sEMG), could be considered to augment the accuracy and comprehensiveness of the assessment. Incorporating these metrics would provide valuable insights into the muscular activity and engagement of the surgeon during the task, potentially enhancing the overall understanding of skill assessment and development. In addition, simplifying and improving our existing measurement system in terms of sensor placement will reduce interference with the microsurgical anastomosis procedure during our next studies.

Furthermore, increasing the sample size of the participants could enhance the generalizability and robustness of the study. In addition, our future studies will include expert surgeons to achieve a broader spectrum of proficiency levels. We will also consider years of experience and training in a longitudinal study for possible analysis.

### Conclusion and outlook

Our study offered a number of metrics in support of an objective skill assessment. Such an objective skill assessment approach may represent a significant improvement over the ALI scoring system in several key aspects. Firstly, it offers objective and quantitative measurements, eliminating the subjective nature inherent in ALI’s error-based scoring. This enhanced accuracy and precision results in a more reliable evaluation of surgical skills. Furthermore, it may lead to a systematic and comprehensive assessment of surgical performance, going beyond error identification to consider factors such as time efficiency, instrument kinematics, and tactile skills.

Secondly, quantitative assessment of skills can support the design of metrics to assess areas requiring skill improvement. In this study, we saw that metrics such as motion smoothness and bimanual dexterity can be good candidates to enable the identification of specific areas for development. This differs from ALI’s emphasis on error scoring and provides actionable insights for enhancing skills. Lastly, our study addresses a fundamental aspect of microsurgical skills by incorporating tactile pressure sensing, which is not measured by ALI. This inclusion further broadens the evaluation process, facilitating a more well-rounded assessment of skill and skill development.

## Data Availability

Data used in this study could be made available by request.

## Code availability

The analysis code used in this study could be made available by request.
